# Data of the first *de novo* transcriptome assembly of the inflorescence of *Curcuma alismatifolia*

**DOI:** 10.1016/j.dib.2018.07.038

**Published:** 2018-07-24

**Authors:** Sima Taheri, Thohirah Lee Abdullah, Yusuf Muhammad Noor, Hirzahida Mohd Padil, Mahbod Sahebi, Parisa Azizi

**Affiliations:** a Department of Crop Science, Faculty of Agriculture, Universiti Putra Malaysia, 43400 Serdang, Selangor, Malaysia; b Laboratory of Bioinformatics and Computational Biology, Malaysia Genome Institute, Jalan Bangi, 43000 Kajang, Selangor, Malaysia; c Laboratory of Climate-Smart Food Crop Production, Institute of Tropical Agriculture and Food Security, Universiti Putra Malaysia, 43400 Serdang, Selangor, Malaysia; d Laboratory of Plantation Science and Technology, Institute of Plantation Studies, Universiti Putra Malaysia, 43400 Serdang, Selangor, Malaysia

**Keywords:** *Curcuma alismatifolia*, *De novo*, Illumina, RNA-Seq, Transcriptome assembly

## Abstract

*Curcuma alismatifolia,* is an Asian crop from Zingiberaceae family, popularly used as ornamental plant in floriculture industry of Thailand and Cambodia. Different varieties with a wide range of colors can be found in species. Until now, few breeding programs have been done on this species and most commercially important cultivars are hybrids that are propagated vegetatively. In spite of other flowering plants, there is still lack of transcriptomic-based data on the functions of genes related to flower color in *C. alismatifolia*. The raw data presented in this article provides information on new original transcriptome data of two cultivars of *C. alismatifolia* by Illumina Hiseq. 4000 RNA-Seq technology which is the first ever report about this plant. The data is accessible via European Nucleotide Archive (ENA) under project number PRJEB18956.

**Specifications Table**

**Table:** 

Subject area	Plant Biology
More specific subject area	Transcriptomics
Type of data	Transcriptome data
How data was acquired	cDNA sequencing was performed using Illumina HiSeq. 4000
Data format	Raw sequences (FASTQ)
Experimental factors	Two samples included purple and white color bracts inflorescences
Experimental features	Fresh and healthy rhizomes of two cultivars were imported from Thailand and were planted in Screen house at Universiti Putra Malaysia, Malaysia. During full blooming, inflorescences were harvested for RNA extraction. Total RNA of colorful and colorless bracts inflorescences were extracted through optimized protocol and were sent to the company for RNA-Seq technology.
Data source location	Universiti Putra Malaysia, Malaysia
Data accessibility	Raw FASTQ files are accessible in European Nucleotide Archive (ENA) under project number PRJEB18956 (http://www.ebi.ac.uk/ena/data/view/PRJEB18956)

**Value of data**

• The data obtained using Illumina sequencer is the first transcriptome data that can be useful for other ornamental ginger breeders.
• The data presented here can be used by other researches for identification of differentially expressed genes (DEGs) and different pathways that may play a significant role in putative gene(s) discovery.
• Further analysis of these data will be applicable for specific simple sequence repeats (SSRs) and single nucleotide polymorphism (SNP) markers development to perform phylogenetic analysis in breeding programs studies of Curcuma genus.

## 1 Data

The dataset of this article provides information about the inflorescence transcriptomic data for two cultivars of *Curcuma alismatifolia* namely ‘Chiang Mai Pink’ and ‘UB Snow 701’ with purple and white bract color generated from the polyA-enriched cDNA libraries prepared from the total RNA extracted using Illumina HiSeq. 4000 platform is provided.

## 2 Experimental design, materials and methods

The rhizomes of two cultivars of *C. alismatifolia* were provided from the *Curcuma* Nursery (Ubonrat), Thailand. Rhizomes were grown in screen house at field 2, Universiti Putra Malaysia, Malaysia. The inflorescences of two cultivars were harvested at the full-bloom stage and were immediately stored at −80 °C until RNA extraction.

Total RNA was isolated from the purple and white bracts of the inflorescences using the modified TRIzol method [1]. The concentration and purity of isolated RNA were determined using NanoDrop 2000 (Thermo Fisher Scientific Inc.). The quality was verified by electrophoresis on 1.5% agarose gel. The two total RNAs were sent to Beijing Genomic Institute (BGI) Company (Shenzhen, China) for the construction of cDNA libraries using mRNA fragments as templates according to the manufacturer׳s instructions. The sequencing of two samples was performed using Illumina HiSeq. 4000 system.

After sequencing, firstly, raw reads were filtered for low-quality, adaptor-polluted, high content of unknown base (N) reads, empty reads, non-coding RNA (such as rRNA, tRNA and miRNA) to get clean reads. After filtering, clean reads were stored in FASTQ format [2]. A total of 131.93 Mb good quality reads were obtained after the removal of low-quality reads. The transcripts of length 200 bp and above were retained for further analysis. Using Trinity (v2.0.6) [3] clean reads were assembled into 65,539 and 80.206 transcripts with GC percentage of 47.27 and 47.22 reaching a total length of 50,262,409 and 64,588,299 for ‘Chiang Mai Pink’ and ‘UB-Snow 701’ cultivars, respectively. The transcripts length ranged from 200 to over 3000 bp, with an average of 766 and 805 bp and an N50 of 1250 and 1345 bp for ‘Chiang Mai Pink’ and ‘UB-Snow 701’ cultivars, respectively (Table 1).

**Table 1:** Statistics of sequencing reads and transcripts of the RNA-Seq generated for ‘Chiang Mai Pink’ (CMP) and UB Snow 701’ (UBS).

**Features**	**CMP**	**UBS**
Total Raw Reads (Mb)	69.97	69.97
Total Clean Reads (Mb)	65.82	66.11
Total Clean Bases (Gb)	6.58	6.61
Clean Reads Q20 (%)	99.06	98.94
Clean Reads Q30 (%)	96.73	96.33
Clean Reads Ratio (%)	94.07	94.48
Total Number of transcripts	65,539	80,206
Total Length (bp)	50,262409	64,588299
Mean Length (bp)	766	805
N50 value	1250	1345
GC(%)	47.27	47.22

N50: a weighted median statistic that 50% of the Total Length is contained in transcripts great than or equal to this value. GC (%): the percentage of G and C bases in all transcripts. Q20: the rate of bases which quality is greater than 20.
