# Supplementary material for: Data of the first de novo transcriptome assembly of the inflorescence of Curcuma alismatifolia
Source: Data Brief. 2018 Jul 24;19:2452–4. doi: 10.1016/j.dib.2018.07.038 (PMC6141965; doi:10.1016/j.dib.2018.07.038)
Supplement: Supplementary file 1 — Supplementary material [file mmc1.docx]

Conflict of Interest and Authorship Conformation Form

Please check the following as appropriate:

- All authors have participated in (a) conception and design, or analysis and interpretation of the data; (b) drafting the article or revising it critically for important intellectual content; and (c) approval of the final version.
- This manuscript has not been submitted to, nor is under review at, another journal or other publishing venue.
- The authors have no affiliation with any organization with a direct or indirect financial interest in the subject matter discussed in the manuscript

**Author’s name Affiliation**

Sima Taheri Department of Crop Science, Faculty of Agriculture, Universiti Putra Malaysia, 43400 Serdang, Selangor, Malaysia


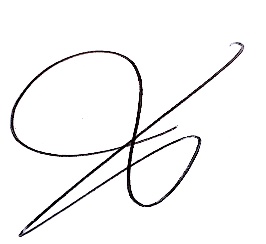


Sima Taheri
